# High expression of HSP47 in ulcerative colitis-associated carcinomas: proteomic approach

**DOI:** 10.1038/sj.bjc.6605163

**Published:** 2009-07-14

**Authors:** K Araki, T Mikami, T Yoshida, M Kikuchi, Y Sato, M Oh-ishi, Y Kodera, T Maeda, I Okayasu

**Affiliations:** 1 Department of Cellular and Histo-pathology, Kitasato University Postgraduate School of Medical Sciences, Sagamihara, Kanagawa, Japan; 2 Department of Pathology, Kitasato University School of Medicine, Sagamihara, Kanagawa, Japan; 3 Department of Molecular Diagnostics, Kitasato University School of Allied Health Sciences, Sagamihara, Kanagawa, Japan; 4 Department of Physics, Laboratory of Biomolecular Dynamics, Kitasato University School of Science, Sagamihara, Kanagawa, Japan

**Keywords:** HSP47, ulcerative colitis, adenocarcinoma, collagen, proteomics

## Abstract

**Background:**

Ulcerative colitis (UC) is a chronic relapsing inflammatory bowel disease, known to be associated with a markedly increased risk of colorectal carcinoma development.

**Methods:**

Using proteomic analysis with two-dimensional gel electrophoresis and mass spectrometry, differentially expressed proteins were assessed between UC-associated cancer and sporadic colon cancer cell lines. Western blot and immunostaining were performed for confirming the expression.

**Results:**

Heat-shock protein of 47 kDa (HSP47) was identified as one of the proteins expressed more highly in UC-associated cancer cell lines, and an immunohistochemical examination confirmed significantly higher levels of HSP47 in UC-associated colon cancers than in sporadic counterparts, the expression increasing with a progression of neoplastic lesions. Heat-shock protein of 47 kDa was further found to be coexpressed with type I collagen in the cytoplasm, and both HSP47 and type I collagen were released from cultured cells into the culture medium.

**Conclusion:**

These results suggest that overexpression of HSP47 is a unique characteristic of UC-associated carcinoma related to type I collagen synthesis, with possible clinical applications.

Recently, carcinogenesis pathways driven by chronic inflammation have been proposed, with ulcerative colitis (UC)-associated colorectal cancer as one very concrete example (Wong and Harrison, 2001; Okayasu *et al*, 2002). Ulcerative colitis features chronic, relapsing, and debilitating idiopathic inflammation of the colon mucosa and submucosa, and patients with long-standing UC are at high risk of neoplastic development. Ulcerative colitis-associated carcinomas are often difficult to detect endoscopically and to discriminate from inflammatory regenerative epithelium (Sada *et al*, 2004; Fujii *et al*, 2008); hence, a molecular marker is urgently required. Recently, proteomics for large-scale studies of gene expression at the protein level have been viewed as a promising experimental approach to explore the control of biological processes and pathways (Lawrie *et al*, 2001; Dundas *et al*, 2005; Kuruma *et al*, 2005). In this study, using UC-associated cancer cell lines and sporadic colorectal cancer cell lines established by our group (Yamashita *et al*, 2004), we sought to determine proteins expressed differently between the two cell lines, using proteomics methods. Agarose two-dimensional gel electrophoresis (agarose 2-DE) and liquid chromatography-tandem mass spectrometry (LC-MS/MS) allowed the detection of heat-shock protein of 47 kDa (HSP47) as one protein showing an overexpression in UC-associated cancer cell lines. Heat-shock protein of 47 kDa is a molecular chaperone that recognises collagen triple helices in the endoplasmic reticulum (ER) (Nagata *et al*, 1986; Nakai *et al*, 1992). Usually, its expression is closely related to that of collagen in collagen-secreting cells, such as fibroblasts. In this study, this expression was further explored in cancer cell lines and immunohistochemically in colon cancer tissues.

## Materials and methods

### Cell culture

Ulcerative colitis-associated cancer cell lines, UCCA-3, UCCA-21, and UCCA-24, and sporadic colorectal cancer cell lines, KE-24, KE-43P, and KE-43W, were earlier established in our laboratory (Yamashita *et al*, 2004). Cell lines UCCA-3, UCCA-21, UCCA-24, KE-43P, and KE-43W were cultured in Dulbecco's modified Eagle's medium (Gibco, Carlsbad, CA, USA), and cell line KE-24 was cultured in RPMI medium 1640 (Gibco), supplemented with 10% fetal bovine serum and 1% penicillin–streptomycin (Gibco), at 37 °C in an atmosphere of 5% CO_2_ and 95% air in a humidified incubator. When the cells reached the logarithmic growth phase, they were washed with phosphate-buffered saline (PBS), scraped off into a tube, briefly centrifuged, quickly frozen in liquid nitrogen, and stored at −80 °C until use. Primary cultures of human fibroblasts were kindly provided by Dr Y Kuroyanagi (R&D Center for Artificial Skin, School of Allied Health Sciences, Kitasato University, Kanagawa, Japan) and similarly cultured in Dulbecco's modified Eagle's medium (Gibco).

### Protein extraction

The cell pellets were sonicated in an extraction solution containing 7 M urea, 2 M thiourea, 0.1 M dithiothreitol, 2.5% pharmalyte (pH 3–10), 2% CHAPS, and Complete Mini EDTA-free (protease inhibitors, Roche Diagnostics, Mannheim, Germany). After centrifugation at 15 000 r.p.m. for 20 min, the supernatant was recovered and the protein concentration was measured using a DC Protein Assay kit (Bio-Rad Laboratories, Hercules, CA, USA).

### Proteomic analysis

Proteomic analysis was carried out using agarose 2-DE according to previously published protocols by Oh-Ishi *et al* (2000) (Tomonaga *et al*, 2004). The agarose isoelectric focusing gel for the first dimension (180 mm in length and 3.4 mm in inner diameter) was prepared by stratification of three types of pharmalyte (pH 2.5–5 for acidic, pH 3–10 and pH 4–6.5 for neutral, and pH 8–10.5 for basic solution). The second dimension for SDS–PAGE used 12% polyacrylamide gels (200 × 130 × 1.3 mm). Cell lysates, equivalent to 500 *μ*g of extracted protein, were subjected to all steps of 2-DE, including agarose isoelectric focusing SDS–PAGE, Coomassie Brillant blue staining, and quantitative analysis using Scion Image (Windows version of public domain NIH image program) (Scion Corporation, Frederick, MD, USA). Protein spots with more than two-fold differences in protein intensity were cut from gels and digested with trypsin to produce unique sets of tryptic fragments. The masses of these fragments were determined using the LC-MS/MS system, consisting of an HPLC system (Nanospace SI-2, Shiseido Fine Chemicals, Tokyo, Japan) and an ion-trap mass spectrometer (LCQ-Deca, Thermo Finnigan, San Jose, CA, USA). A minimum score of 80 was selected as the criterion for the positive identification of proteins. We used the database of the SEQUEST program (an attached software to the LQC-Deca, Thermo Finnigan, San Jose, CA, USA) to identify proteins from measured masses of tryptic peptides and their MS/MS fragments.

### Western blot analysis

Protein samples (5 *μ*g) were separated by SDS–PAGEelectrophoresis using 10% gels, and blotted onto Hybond-P, PVDF membranes (Amersham Pharmacia Biotech, Buckinghamshire, UK). Immunoassays were performed using anti-HSP47 (monoclonal, SPA-470, 1:10 000, Stressgen Biotechnologies, Ann Arbor, MI, USA) and anti-*β*-actin (monoclonal, AC-15, 1 : 10 000, Sigma, St Louis, MO, USA) antibodies. The membranes were then incubated with anti-mouse immunoglobulin-HRP (DakoCytomation, Glostrup, Denmark), and protein bands were visualised using enhanced chemiluminescence (Amersham Pharmacia Biotech) according to the manufacturer's manual.

### Immunocytochemical staining

The harvested cells were washed and re-suspended in PBS and centrifuged at 1000 r.p.m. for 5 min using Cytospin 2 (Thermo Fisher Scientific Inc., Waltham, MA, USA). Cells on slide glasses were immediately immersed in 95% ethanol, and immunocytochemical staining was performed using the labelled polymer–peroxidase complex method (EnVision+ System HPR; DakoCytomation). Endogenous peroxidase was blocked using 0.3% hydrogen peroxide for 30 min. After incubation with Protein Block Serum-Free (DakoCytomation), sections were incubated overnight with an anti-HSP47 antibody (monoclonal, SPA-470, 1 : 1000, Stressgen Biotechnologies) at 4 °C. After incubation with a labelled polymer (anti-mouse, EnVision+ System HPR; DakoCytomation) for 10 min at room temperature, 3,3′-diaminobenzidine was used as the chromogen. Nuclei were counter-stained with 0.3% methylgreen solution.

### Immunohistochemical staining

From the pathology files of the Kitasato University Hospital and the Kitasato University East Hospital, cases of 63 UC-associated lesions and 81 sporadic colon tumour lesions were collected. Given that almost all UC-associated neoplastic lesions were located at the left hemicolon, sporadic colon tumours were mainly collected from cases of the left hemicolon. The clinicopathological data of the cases included in this study are summarised in Table 1 . Formalin-fixed paraffin-embedded tissue blocks of specimens of 16 UC-associated low-grade dysplasias (LGDs), 12 high-grade dysplasias (HGDs), 10 invasive adenocarcinomas, and 25 areas of inflammatory regenerative mucosa, and of 17 cases of sporadic tubular adenoma with LGD, 16 cases of sporadic tubular adenoma with HGD, and 48 cases of sporadic invasive adenocarcinoma were then collected. In addition, 48 normal control mucosa specimens collected at least 5-cm distant from sporadic cancer lesions were also examined. Serial 4-*μ*m-thick sections were cut for immunohistochemical staining using an EnVision+ kit (EnVision+ System HPR; DakoCytomation) and anti-HSP47 antibodies at the working dilution (SPA-470, 1 : 200, Stressgen Biotechnologies). Endogenous peroxidase was blocked by 0.3% hydrogen peroxide for 30 min. After incubation with Protein Block Serum-Free (DakoCytomation), sections were incubated overnight with anti-HSP47 antibody at 4 °C. After incubation with a labelled polymer (anti-mouse, EnVision+ System HPR; DakoCytomation) for 30 min at room temperature, 3,3′-diaminobenzidine was used as the chromogen. Nuclei were counter-stained using Mayer's haematoxylin to facilitate histopathological assessment. Immunoreactivity for HSP47 was evaluated using the scoring system as previously described (Tokuyama *et al*, 2007), combining staining intensity (none, 0; weak, 1; moderate, 2; intense, 3) with percentages of positive cells (0, <1%; 1, 1–25%; 2, 25–50%; 3, 50–75%; 4, >75%).

### Double immunofluorescence staining of cell lines

A UC-associated cancer cell line (UCCA-3) and a sporadic carcinoma cell line (KE-24) were cultured on chamber glass slides (Thermo Fisher Scientific Inc.), fixed with 50% methanol and 50% acetone for 5 min, and permeabilised in 0.1% Triton X-100 in PBS for 5 min. The slides were then incubated with anti-monoclonal HSP47 antibody (SPA-470, 1:200, Stressgen Biotechnologies) for 1 h at room temperature. After washing, anti-collagen type I polyclonal antibodies (COL-1, 1 : 500, Sigma) were applied for 1 h at room temperature. The secondary antibodies were rhodamin-labelled anti-mouse IgG and fluorescein isothiocyanate (FITC)-labelled anti-rabbit IgG (both 1 : 500, Molecular Probes, Leiden, The Netherlands), for 30 min at room temperature. Cell nuclei were counter-stained with DAPI. Negative control staining was performed by omitting the primary antibody incubation. Immediately after treatment, the cell lines were observed under a fluorescence microscope.

### ELISA for measuring type I collagen concentrations in culture media

Cell lines UCCA-3, UCCA-21, UCCA-24, KE-24, KE-43P, and KE-43W were cultured in 24-well plates with 1 ml of the culture medium. A human collagen type I ELISA kit (Applied Cell Biotechnologies Inc., Yokohama, Japan) was used to measure the concentration of type I collagen in the culture medium. When the cells reached confluence, 0.2 ml of the culture medium and 0.1 ml of pepsin solution (pepsin 0.6 mg ml^−1^ dissolved in 150 mM acetic acid) were mixed, and collagen was digested overnight at 4 °C. Biotinylated anti-type I collagen antibodies were then added and 50*μ*l aliquots of the mixture were applied to microplate wells on which type I collagen was immobilised. After incubation for 1 h at room temperature and washing, an avidin-horseradish peroxidase conjugate solution was added to the wells, followed by incubation for 1 h at room temperature. After washing, 50 *μ*l of substrate solution was added, and after incubation for 15 min at room temperature, 50 *μ*l of stop solution (1 N H_2_SO_4_) was added. The concentration of collagen type I was obtained by measuring absorbance at 450 nm on a microplate reader.

### Western blotting of HSP47 in cell culture medium

Cell lines were cultured in 12-well plates in 2 ml of the culture medium. When they reached confluence, the culture medium was replaced with a serum-free BIO-RICH 1 medium (MP Biomedicals, Irvine, CA, USA) for 4 days. Samples were collected, and 3 ml of extract solution (7 M urea, 2 M thiourea, 0.1 M dithiothreitol) was added for overnight incubation at 4 °C. After the solutions were concentrated by acetone precipitation, HSP47 was detected by western blot analysis. As a negative control, the culture medium alone was used.

### Statistical analysis

All statistical tests were conducted using StatView 5.0 software (SAS institute Inc., Cary, NC, USA). Comparisons among more than two groups were assessed using the Kruskal–Wallis test with the Mann–Whitney *U*-test as a *post hoc* test. Comparisons between two groups were made using the Mann–Whitney *U*-test. Statistical significance was concluded at *P*<0.05.

## Results

### Proteomic analysis

**Figure 1:**
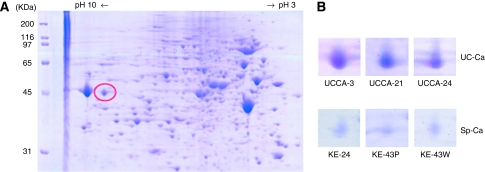
Agarose two-dimensional electrophoresis gel patterns of cell lines. (**A**) Representative proteome maps derived from the UCCA-3, UC-associated cancer cell line. The red circle indicates the protein spot, HSP47. (**B**) The zoomed two-dimensional electrophoresis gel images show expression of HSP47. UC-associated cancer cell lines show an increased expression of HSP47 compared with sporadic cancer cell lines (Coomassie blue-stained two-dimensional electrophoresis gels).

Representative agarose 2-DE images are shown in Figure 1A. A total of more than 2000 spots were obtained, 67 being differentially expressed between UC-associated cancer and sporadic colorectal cancer cell lines. Of the 67 spots, 2 protein spots showed a more than 2-times higher expression and 4 spots showed a more than 2-times lower expression in all 3 UC-associated cancer cell lines than in all 3 sporadic cancer cell lines. On tryptic digestion and LC-MS/MS system analysis, six proteins were identified. Heat-shock protein of 47 kDa was detected as one protein consistently expressed higher in three UC-associated cancer cell lines than in three sporadic colorectal cancer cell lines (Figure 1B). Another protein that showed a higher expression in UC-associated cell lines was a cytoskeletal protein. Four proteins that showed a higher expression in sporadic cancer cell lines were a cytoskeletal protein, a signal transduction factor, and two enzymes associated with acetyl-coA metabolism and folate metabolism. In addition, HSP70 was detected as a protein that showed a tendency to increase in two UC cell lines, significant difference not being recognised.

### HSP47 expression by western blot analysis and immunocytochemical staining

**Figure 2:**
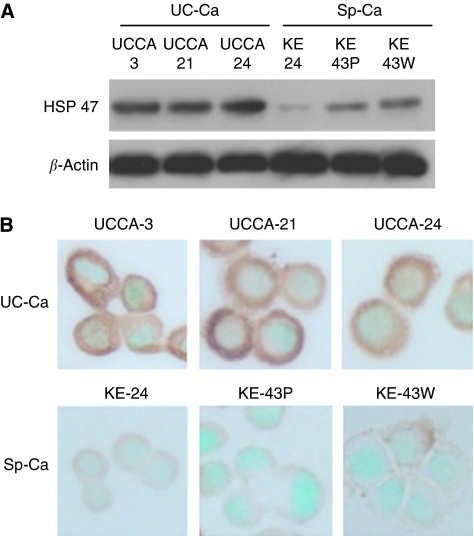
Western blot analysis of the HSP47 expression in cell lines. (**A**) UC-associated cancer cell lines (UC-Ca) and sporadic cancer cell lines (Sp-Ca). *β*-Actin expression was used as a control. (**B**) Immunocytochemical staining of HSP47 in the six cell lines. The strong reactions in UC-associated cancer cell lines is noted.

Upregulation of HSP47 in UC-associated cancer cell lines by proteomics analysis was further confirmed using immunoblotting (Figure 2A) and immunocytochemical staining (Figure 2B). With immunocytochemical staining, the HSP47 protein was expressed in the cytoplasm of the cells.

### HSP47 expression in UC-associated and sporadic colon tumours

**Figure 3:**
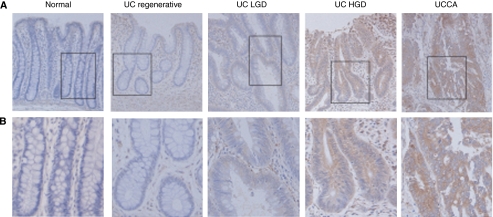
Immunohistochemical staining of HSP47 in tissues. (**A**) UC-associated lesions and normal mucosa. Normal epithelial cells are not stained for HSP47. It is noted that the increased expression of HSP47 with progression through to carcinoma is apparent. (**B**) A higher magnification of the boxed area is shown in panel A. UCLGD, UC low-grade dysplasia; UCHGD, UC, high-grade dysplasia; UCCA, UC-associated adenocarcinoma.

**Figure 4:**
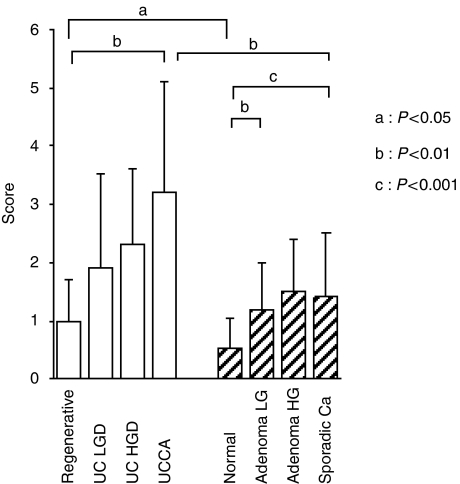
The epithelial expression of HSP47 in UC-associated (open bars) and sporadic colon tumour lesions (hatched bars). Data are mean scores and s.d. (a) *P*<0.05; (b) *P*<0.01; and (c) *P*<0.001; LGD, low-grade dysplasia; HGD, high-grade dysplasia.

Typical HSP47 immunohistochemical staining patterns of UC-associated lesions are shown in Figure 3. The expression of HSP47 was lacking or very weak in normal epithelial cells. In tumour tissue, staining was evident in the cytoplasm of epithelial tumour cells. Both UC-associated and sporadic colon tumours showed a significantly higher expression than did normal mucosa. In addition, HSP47 tended to gradually increase from low-grade intramucosal lesions, dysplasia, and adenoma towards invasive carcinoma, in both series. Furthermore, UC-associated carcinoma showed a higher expression than did sporadic colonic carcinoma (*P*<0.01), and UC-regenerative mucosa showed a significantly higher expression than did normal mucosa (*P*<0.05) (Figure 4). When UC-regenerative mucosae were histologically classified into active and inactive UC groups, there was no difference in expression between the two groups (active UC, 1.0±0.7; inactive UC, 1.0±0.8).

### Immunofluorescent double staining of HSP47 and type I collagen

**Figure 5:**
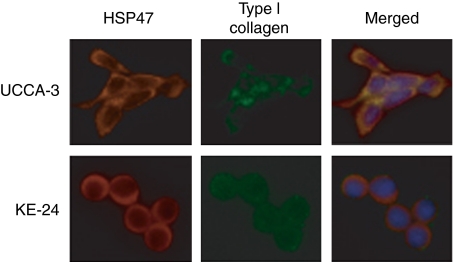
Double immunofluorescence staining for HSP47 (red-rhodamin) and type I collagen (green-FITC) in a UC-associated cancer cell line (UCCA-3) and a sporadic cancer cell line (KE-24). The cells were counterstained with DAPI to visualise the nuclei.

Immunofluorescent double staining of HSP47 and type I collagen showed co-localisation in UC-associated cancer cell lines (Figure 5). In contrast, no or only a weak expression of type I collagen was found in sporadic colorectal cancer cell lines.

### Type I collagen synthesis measured by ELISA

**Figure 6:**
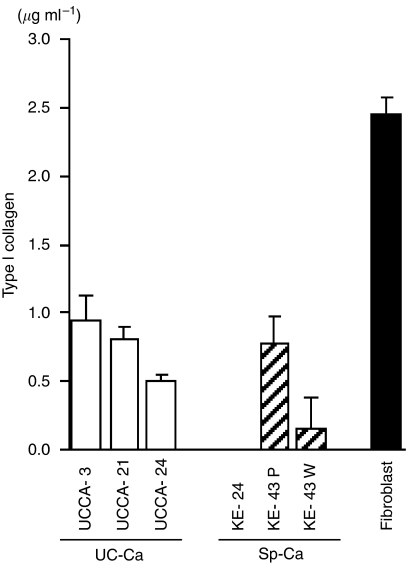
Collagen synthesis of UC-associated cancer cell lines (UC-Ca) (open bars), sporadic cancer cell lines (Sp-Ca ) (hatched bars), and human fibroblast cells (black bars). Data are given as concentrations of type I collagen in medium. Vertical lines show s.d.

Type I collagen synthesis was shown in both UC-associated and sporadic colorectal cancer cell lines, as well as in human fibroblasts. However, compared with UC-associated cancer cell lines, collagen synthesis was at a low level in the KE 24 and KE43W cell lines (Figure 6).

### HSP47 in culture medium

**Figure 7:**
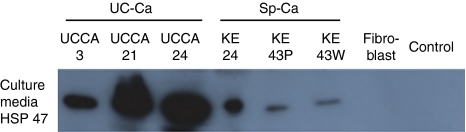
Western blot of HSP47 in cell culture medium. An increased release of HSP47 was shown in UC-associated cancer cell lines than in sporadic colorectal cancer cell lines.

HSP47 was detected by immunoblotting in the culture media, particularly from UC-associated cancer cell lines. There was an extremely limited release from normal human fibroblasts (Figure 7).

## Discussion

Proteomic analysis of UC-associated cancer cell lines and sporadic colorectal cancer cell lines in this study showed an upregulated expression of the ER stress-response protein HSP47 in UC-associated cancer cell lines. In the culture environment, as neither inflammation nor influence of stroma exists, protein expression is limited. Therefore, we confirmed the expression using clinical materials by immunohistochemical staining. The expression of HSP47 increased with progression through dysplasia to UC-associated carcinoma. Heat-shock protein of 47 kDa is a collagen-binding, stress-inducible protein localised in the ER that participates in the intracellular processing, folding, assembly, and secretion of procollagens, especially in collagen-secreting cells, such as fibroblasts, for example, in fibrotic lesions (Razzaque and Taguchi, 1999). On the other hand, in epithelial or carcinoma cells, few reports have described the expression of HSP47. Our results are in line with earlier reports of overexpression of HSP47 in pancreatic carcinoma (Maitra *et al*, 2002), gastric carcinoma (Hirai *et al*, 2006), and head and neck squamous cell carcinoma (Li *et al*, 2008). Another chaperone protein, mortalin (mitochondrial heat-shock protein 70), was reported as the protein that contributed to colorectal adenocarcinomas (Dundas *et al*, 2005). Heat-shock protein of 47 kDa has an important role in the biosynthesis of procollagens, and co-localisation of HSP47 and collagen type I could be shown by double immunofluorescent staining in this study. In tissue, although HSP47 was found to be expressed in both epithelial and stromal cells within cancers, only the former showed an increase with progression to greater malignancy. A significantly higher expression in UC-associated than in sporadic carcinomas suggests that the epithelial expression of HSP47 may be influenced by background inflammation. However, as upregulation was also noted in cultured UC-associated cancer cell lines, the increase may be an intrinsic property.

In culture media, type I collagen was detected. Therefore, not only fibroblasts but also epithelial cancer cells produce type I collagen. Although the functions of collagen from epithelial cells are not clear, they may reflect stromal collagen metabolism. From the literature, inflammatory cytokine production during chronic inflammation may lead to excessive collagen deposition and eventually to fibrosis in UC (Lawrance *et al*, 2001a, 2001b). Metalloproteinases may also be overexpressed and degrade stromal collagen with inflammatory bowel disease (von Lampe *et al*, 2000; Stumpf *et al*, 2006). Therefore, in chronic inflammatory condition, the biosynthesis of collagen might need to be increased chronically, with collagen produced by epithelial cells supplementing that from fibroblasts.

Heat-shock protein of 47 kDa clearly has the potential to be a biomarker for UC-associated cancer. Although the mechanism of release from cancer cell remains to be clarified, cell death could be involved. Alternatively, a deletion of the ER retention signal or an overflow of HSP47 from the ER retention signal may cause a leakage of HSP47 to outside the cell (Yokota *et al*, 2003). Only very limited release was noted from cultured fibroblasts. Recently, high-serum auto-antibodies to HSP47 or HSP47 itself have been reported under inflammatory conditions, such as interstitial pneumonia and mixed connective tissue disease (Yokota *et al*, 2003; Kakugawa *et al*, 2008). If HSP47 is released from cancers in patients with UC, its detection in serum would be helpful for the clinical detection of malignancy. As surveillance by endoscopic examination sometimes fails to detect cancer lesions (Fujii *et al*, 2008), an easily measurable biomarker would clearly be very welcome.

In conclusion, overexpression of HSP47 is a unique characteristic of UC-associated carcinoma. Further studies are required to elucidate its clinical significance.

## Figures and Tables

**Table 1:** Clinicopathological data of the patients

					**Carcinoma**	
	**Age (years) (mean±s.d.)**	**Gender (M : F)**	**Duration from the onset of UC (years) (mean±s.d.)**	**Site (R : L)**	**Differentiation (well : mod-poor)**	**Stage^a^ (I : II : III : IV)**
*UC-associated lesions*						
UC regenerative mucosa	39.1±14.2	13 : 13	6.4±5.4	1 : 25		
UC low-grade dysplasia	37.5±15.1	6 : 9	9.7±2.5	0 : 16		
UC high-grade dysplasia	42.3±16.1	10 : 2	12.1±4.0	0 : 12		
UC-associated Ca	33.4±14.6	3 : 6	9.5±4.4	0 : 10	3 : 7	1 : 2 : 7 : 0
						
*Sporadic colon tumour*						
Normal mucosa	63.3±12.3	26 : 22		0 : 48		
Adenoma, low grade	57.8±14.3	13 : 3		0 : 15		
Adenoma, high grade	69.2±9.0	10 : 5		0 : 15		
Adenocarcinoma	63.3±12.3	26 : 22		0 : 48	18 : 30	3 : 13 : 32 : 0

Ca=carcinoma; F=female; L=left; M=male; mod-poor=moderate to poor; R=right; UC=ulcerative colitis.

a According to Unio Internationalis Contra Cancrum (UICC) criteria (Sobin and Wittekind, 2002).
